# Commentary: Comparison of different therapeutic approaches for pulmonary cryptococcosis in kidney transplant recipients: a 15-year retrospective analysis

**DOI:** 10.3389/fmed.2023.1275042

**Published:** 2023-10-17

**Authors:** Lei Gu, Jing Lin, Wei Liu, Jian Yue, Jian-an Huang

**Affiliations:** ^1^Department of Pulmonary and Critical Care Medicine, The First Affiliated Hospital of Soochow University, Suzhou, China; ^2^Institute of Respiratory Diseases, Soochow University, Suzhou, China; ^3^Suzhou Key Laboratory for Respiratory Diseases, Suzhou, China; ^4^Department of Infectious Diseases, The First Affiliated Hospital of Soochow University, Suzhou, Jiangsu, China; ^5^Department of Respiratory and Critical Care Medicine, The 900th Hospital of Joint Logistic Support Force, People's Liberation Army, Fujian Medical University, Fuzhou, Fujian, China; ^6^The People's Hospital of Gaozhou, Gaozhou, China

**Keywords:** pulmonary cryptococcosis, pulmonary nodules, lung cancer, cryptococcus antigen, nodule

We read with great interest the article by Chen et al. (1) published in *Front. Med. (Lausanne)*. The remarkable discovery of this study is that patients with pulmonary cryptococcosis (PC) often received surgical resection as a therapeutic approach (39.13%, 9/23) because PC frequently manifested nodule-shaped lesions, which closely resemble lung carcinoma. In a prospective multi-center clinical study of HIV-negative PC in China, the majority of the patients were devoid of underlying diseases (70.24%, 321/457) and were immunocompetent (87.75%, 401/457), and 25.16% (115/457) of the patients had no clinical symptom or physical signs (2). Compared with immunocompromised patients, 57.14% of immunocompetent patients were asymptomatic (3). The chest computed tomography (CT) scans revealed that the majority of PC lesions presented as solitary or multiple nodules, constituting 51.54% middle-sized nodules (ranging from 1 to 5 cm), 28.07% small-sized nodules (ranging from 3 mm to 1 cm) (2), and 10.09% nodular masses exceeding 5 cm (4). These intricate presentations often give rise to a diagnostic challenge, with cases frequently being initially misinterpreted as instances of lung cancer. The misdiagnosis rate of PC during the initial visit was even 43.42% (33/76), often as cancer by false-positive 18 FDG-PET (28 out of 46 cases) (5). Therefore, early diagnosis of nodular or mass PC remains crucial, which can not only avoid misdiagnosis and unwarranted surgical procedures but also reduce potential harm to patients and healthcare costs.

Currently, diagnosis of PC mainly relies on histopathological examinations (74.40%, 340/457) and cryptococcus antigen (CrAg) detection (37.64%, 172/457) (2). Of course, smears and cultures of cryptococcus in respiratory samples, such as sputum or bronchoalveolar lavage fluid (BALF), are also important diagnostic methods. However, the proportion of cases that can be confirmed by smears and cultures of cryptococcus is extremely low. Due to the invasive nature of percutaneous lung biopsy and the difficulty in biopsying some pulmonary nodules, the histopathological diagnosis of PC is limited, which often leads to missed diagnoses and misdiagnoses, causing unwarranted surgical operations. CrAg detection has become an important method for the clinical diagnosis of PC. In HIV-negative PC patients, the sensitivity of the serum CrAg test was 71.99% (203/282) (2). The sensitivity of serum CrAg was higher in HIV patients with PC (6). A meta-analysis evaluated the diagnostic accuracy of the CrAg lateral flow immunoassay (LFA) on serum and found that the pooled sensitivity and specificity values of LFA in serum were 97.6 and 98.1%, respectively (7). Zhu et al. (8) reported that the diagnostic performance of BALF CrAg-LFA for PC was notable, demonstrating a sensitivity of 93.9% (31/33), a specificity of 100% (78/78), and an accuracy of 98.2%, which was superior to the diagnostic value of serum CrAg-LFA. Zeng et al. (9) also found that the sensitivity and specificity of serum CrAg-LFA were 75.0 and 99.6%, respectively, while those of BALF were 93.1 and 100%, respectively. The LFA is a point-of-care test that requires no specimen preparation. It has become the recommended test due to its lower cost and ease of testing. However, the positive rate of CrAg in immunocompetent hosts was lower than that in immunocompromised hosts, and a negative test result could not exclude PC, especially for normal immune function patients.

To sum up, we urge clinicians to consider nodular PC in the differential diagnosis of pulmonary nodules and masses. Clinicians should improve their knowledge of PC imaging features and increase their understanding and attention to the disease, and serum or BALF CrAg screening should be performed in patients who cannot be excluded from PC first.

## Author contributions

WL: Visualization, Writing—review and editing. LG: Writing—original draft. JL: Writing—original draft. JY: Conceptualization, Validation, Writing—review and editing. J-aH: Writing—review and editing.
